# 

**Published:** 1908-09-26

**Authors:** 


### NOTES ON TREATMENT.

**Rheumatoid Arthritis.**

The drugs I have found most useful in the treatment of rheumatoid arthritis are guaiacol and potassium iodide.—*Dr. A. P. Luff.*

**The Therapeutic Value of Water.**

Many people, especially women, limit far too much their consumption of water. They do not take enough to make possible a due metabolism and elimination, and thus waste products probably accumulate in the by-paths of the circulation.—*Dr. W. B. Lees.*

**Threatened Abortion.**

If there is bleeding but no pain, small doses of ergot may be given. If there are pains as well as bleeding, it is better to give small doses of opium. In all cases absolute rest in bed is essential. The patient should be kept strictly in the recumbent position.—*Dr. W. J. Gow.*

**Vertigo.**

As a rule it may be accepted that epileptic vertigo answers to bromide, renal vertigo to iodide of potassium, traumatic vertigo to perchloride of mercury, labyrinthine vertigo to quinine or (if the patient has an idea that quinine upsets him) to small doses of sodium salicylate.—*Dr. Dundas Grant.*

### International Congress on Tuberculosis.

The following are the British delegates to the International Congress on Tuberculosis now being held at Washington from Sept. 21 to Oct. 12 : Dr. A. Wynter Blyth (Royal Sanitary Institute), Dr. F. Bushnell (Devon and Cornwall Sanatorium), Colonel W. Cornwallis-West (Denbighshire County Council), Professor Delépine (Victoria University, Manchester), Dr. C. E. Fitzgerald (Royal College of Physicians of Ireland), Dr. G. A. Gibson (University of Edinburgh, University of St. Andrews), Dr. P. H. S. Hartley, M.V.O. (St. Bartholomew's Hospital, Brompton Hospital, King Edward VII. Sanatorium), Professor M. Hay (University of Aberdeen), Dr. G. A. Heron (Royal Society of Medicine), Professor E. W. Hope (University of Liverpool), Councillor W. Kinmouth (Corporation of Cork), Dr. A. Latham (University of Oxford, St. George's Hospital, Brompton Hospital, Royal Society of Medicine), Mr. F. Link, J.P. (Corporation of London), J. Patten Macdougall (Local Goversment Board of Scotland), Dr. D. J. Mackintosh (University of Glasgow), Dr. J. Miller (University of Birmingham), Mr. T. Munro (County Council of Lanark), Dr. A. Newsholme (Local Government Board of England), Councillor H. O'Shea (Corporation of Cork), Professor W. Osler (University of Oxford, Lister Institute of Preventive Medicine, King Edward VII. Sanatorium), Dr. W. S. Paget-Tomlinson (County Council of Westmorland), Dr. J. J. Perkins (St. Thomas's Hospital), Dr. R. W. Philip (Royal College of Physicians of Edinburgh; Royal Infirmary, Edinburgh), Dr. Nathan Raw (National Association for the Prevention of Consumption, Invalid Children's Aid Association), Dr. J. C. Renton (Faculty of Physicians and Surgeons, Glasgow), Councillor A. Shelmerdine, J.P. (City of Liverpool), Professor W. R. Smith (Royal Institute of Public Health), Dr. J. E. Squire, C.B. (Mount Vernon Hospital for Consumption), Mr. T. J. Stafford, F.R.C.S. (Local Government Board of Ireland), Dr. H. E. Symes-Thompson (Royal Hospital for Diseases of the Chest), Dr. C. Templeman (Town Council of Dundee), Dr. J. C. Thresh (County Council of Essex), Mr. A. W. West (St. George's Hospital), Dr. J. T. Wilson (County Council of Lanark), Dr. Theodore Williams, M.V.O. (Royal College of Physicians, Medical Society of London, King Edward VII. Sanatorium, National Association for the Prevention of Consumption), Professor G. S. Woodhead (University of Cambridge, University of London, Queen Victoria's Jubilee Institute for Nurses).

### ANSWERS TO CORRESPONDENTS.

H. H. L.—The Royal Society of Medicine, incorporated in 1907, consists at the present time of fifteen sections, representing seventeen previously distinct medical societies and associations, of which the oldest was the Royal Medical and Chirurgical Society, founded in 1805. The Medical Society of London on the other hand still continues its separate existence. The Royal Society of Medicine consists of Fellows of the Society and of members of the various sections of the Society. Each section is autonomous and holds regular meetings at the Society's rooms, 20 Hanover Square, London, W. Joint meetings of various sections are also held, as well as meetings of the Society as a whole. There is a very large and complete library of over 80,000 volumes, and the proceedings of the Society are published monthly. The Secretary is Mr. J. Y. W. MacAlister.

J. H. Barham.—The higher certificate of the Cambridge Schools' Examination Board confers exemption from the matriculation examination of the University of London. After next June five and a half years will be the minimum period required for the London M.B., five years for the L.R.C.P. Lond. and M.R.C.S. Eng. (the diplomas of the English Conjoint Board), and five years for the licence in medicine and surgery of the Apothecaries' Society of London. The latter is, and has been for some time, a complete registerable qualification. The best time to enter is undoubtedly October, although it is quite feasible to join the Medical School in April, at the beginning of the summer session.

### THE GRADUATES' MEDICAL SCHOOLS.

DIARY FOR WEEK SEPT. 28 to OCT. 3.

**MEDICAL GRADUATES' COLLEGE AND POLYCLINIC, 22 Chenies Street, W.C.**

**At 5.15 p.m.**

**Oct. 1**, Dr. Robert Jones, **Insanity, Wit, and Humour.**

